# Comprehensive analysis of prognosis-related alternative splicing events in ovarian cancer

**DOI:** 10.1080/15476286.2022.2113148

**Published:** 2022-08-18

**Authors:** Shizhi Wang, Shiyuan Wang, Xing Zhang, Dan Meng, Qianqian Xia, Shuqian Xie, Siyuan Shen, Bingjia Yu, Jing Hu, Haohan Liu, Wenjing Yan

**Affiliations:** Key Laboratory of Environmental Medicine Engineering, Ministry of Education, School of Public Health, Southeast University, Nanjing, Jiangsu, China

**Keywords:** Alternative splicing, prognostic factor, tumour immune microenvironment, clinically applicable model, ovarian cancer

## Abstract

Ovarian cancer (OV) is characterized by high incidence and poor prognosis. Increasing evidence indicates that aberrant alternative splicing (AS) events are associated with the pathogenesis of cancer. We examined prognosis-related alternative splicing events and constructed a clinically applicable model to predict patients’ outcomes. Public database including The Cancer Genome Atlas (TCGA), TCGA SpliceSeq, and the Genomics of Drug Sensitivity in Cancer databases were used to detect the AS expression, immune cell infiltration and IC50. The prognosis-related AS model was constructed and validated by using Cox regression, LASSO regression, C-index, calibration plots, and ROC curves. A total of eight AS events (including FLT3LG|50942|AP) were selected to establish the prognosis-related AS model. Compared with high-risk group, low-risk group had a better outcome (*P* = 1.794e-06), was more sensitive to paclitaxel (*P* = 0.022), and higher proportions of plasma cells. We explored the upstream regulatory mechanisms of prognosis-related AS and found that two splicing factor and 156 tag single nucleotide polymorphisms may be involved in the regulation of prognosis-related AS. In order to assess patient prognosis more comprehensively, we constructed a clinically applicable model combining risk score and clinicopathological features, and the 1 -, and 3-year AUCs of the clinically applicable model were 0.812, and 0.726, which were 7.5% and 3.3% higher than that of the risk score. We constructed a prognostic signature for OV patients and comprehensively analysed the regulatory characteristics of the prognostic AS events in OV.

## 1 Introduction

Ovarian cancer (OV) has the highest mortality rate among gynaecological malignancies, posing a serious threat to female health [1]. It is estimated that 60–70% of OV patients have been diagnosed at stage III/IV, due to the lack of typical symptoms and early screening methods [2]. Despite significant advances in the treatment of OV, especially emerging immunotherapies, more than 75% of patients with advanced OV eventually die from the disease [3,4]. Features currently used for prognosis are histological subtypes, tumour stage, and patient’s age. Nevertheless, OV is clinically heterogeneous. Patients with similar clinicopathological may display different clinical outcomes. Given the genetic heterogeneity of patients, building reliable prognostic prediction models may improve patient outcomes.

To date, several studies have attempted to establish molecular signatures models based on gene expression data to predict patients’ survival and prognosis, including gene expression levels based on mRNA, miRNA, and lncRNA [5–7]. Although these models played an important role in predicting the survival of OV patients, they focused on changes in gene expression levels and ignored the diversity of RNA subtypes with different alternative splicing (AS) regulations. In a recent study, PCAT19-long was found to interact with HNRNPAB to activate a subset of cell-cycle genes, thus promoting prostate cancer (PCa) tumour growth and metastasis. In contrast, the PCAT19-short isoform reduced PCa risk [8]. Nuclear SRSF1 promoted the synthesis of MKNK2b and inhibited the expression of MKNK2a, contributing to the poor prognosis of colon adenocarcinoma patients [9]. Similarly, it was also found in OV that patients with high expression of the ECM1a subtype had a poor prognosis, whereas high expression of the ECM1b had a better prognosis [10]. These results supported that the different isoforms had divergent functional roles. Therefore, changes in gene subtype expression levels also need to be incorporated into studies when constructing predictive models.

As an important post-transcriptional regulatory mechanism, AS regulates gene expression and protein diversity from a limited number of loci [11]. Under pathological conditions, splicing pattern changes lead to the loss or gain of critical protein domains, resulting in alteration function, stability, and subcellular localization [12–14]. Recent trends in different types of cancer research revealed that AS alterations might play important roles in the proliferation, metastasis, and apoptosis of cancer cell [10,15,16]. More importantly, there was increasing evidence that AS could also have a great impact on the microenvironment formation by regulating tumour-associated immune cell infiltration [17]. Therefore, AS-related genes might have new potential in cancer therapy. Emerging evidence had shown that genetic variation, especially single nucleotide polymorphisms (SNPs), could influence the regulation of AS [18,19]. For example, Guo et al. discovered that SNP rs4383 could regulate the AS events of MAFF and was associated with bladder cancer risk [19]. Furthermore, accumulating studies demonstrated that AS events might be intricately regulated by key splicing factors (SFs) [20]. Aberrant expression of SFs leaded to subversive changes in tumour-specific AS events, which influenced carcinogenesis and progression [21]. Therefore, it was of great significance to study the potential prognostic manifestations and regulatory mechanisms of AS in OV.

To date, few studies have comprehensively analysed the clinical significance of AS in OV and its regulatory mechanisms. We integrated AS events and clinical information from TCGA OVs, discerned AS events associated with prognosis in OV, and constructed and validated prognostic risk models. Furthermore, we identified distinct OV clusters based on prognostic risk models, investigated the association between clusters and immune cell infiltration, and screened chemotherapeutic drugs for OV. Finally, the development of SF-AS and SNP-AS regulatory networks revealed potential regulatory mechanisms involved in the prognosis of OV patients.

## 2 Materials and methods

### 2.1 Data resources from public databases

The mRNA data and the AS data of OV were obtained from TCGA (https://tcga-data.nci.nih.gov/) data portal and TCGA SpliceSeq (https://bioinformatics.mdanderson.org/TCGASpliceSeq) database, respectively. The 356 OV patients were selected for further analysis, using PSI (percent splicing) values > 75% as filter values and excluding AS data with more than 30% missing values. The corresponding clinical data were retrieved from the UCSC Xena database (https://Xena.UCSC.edu/).

### 2.2 Identification of prognosis-related AS events

To identify prognosis-related AS events, the univariate Cox regression analysis was performed to determine the relationship between AS events and OS. The prognosis-related AS events based on the seven types of AS events were presented using UpSet plot and volcano plot. In addition, bubble plots were displayed to summarize the top 10 AS events for seven types. AS events with a significant *P* < 0.05 were selected as prognosis-related AS.

### 2.3 AS prognostic risk model establishment and validation

First, all the 356 OV patients were randomly assigned in a ratio of 2:3 to two data sets, including training set (n = 142) and internal validation set 1 (n = 214). Besides, the entire TCGA set constituted validation set 2. Second, the top 20 prognosis-related AS were screened through LASSO Cox analysis and multivariate Cox regression analysis to build a prognostic risk model. The formula of the risk score was calculated as follows: $Riskscore=\overset{n}{\underset{i=1}{\sum }}Coefi*xi$. All patients were divided into a high-risk group and a low-risk group based on the median risk score. The difference in prognosis between the two groups was compared using Kaplan Meier (K-M) curve. Receiver operating characteristic curves (ROC) and the area under the curve (AUC) were performed to evaluate the discriminative power of the prognostic risk model. Finally, the AS prognostic risk model was applied to the validation sets.

### 2.4 Nomograms

To enhance the predictive ability of the model, risk score, age, grade, and tumour stage were integrated into the clinical nomogram. The concordance index (C-index) and calibration curves were applied to evaluate the prediction accuracy between the actual results and the predicted model. Nomogram and calibration plots were drawn through the ‘rms’ package of R software.

### 2.5 Regulatory networks of splicing factor AS events (SF-AS) and genetic variants AS events (SNP-AS)

Splicing factor (SF) is a protein factor that contributed to the splicing process of RNA precursor, which is closely associated with the tumour occurrence and treatment. Therefore, the SF data was obtained from the spliceaid2 database and then the correlation between the expression levels of SF and PSI values of prognosis-related AS events was analysed using Spearman correlation analysis [22]. |R| > 0.3 and P < 0.001 were considered statistically significant. Finally, the SF-AS regulatory network was constructed and visualized by Cytoscape software. SNPs were also involved in the AS of genes. Therefore, we screened SNPs associated with prognosis-related AS events from the CancerSplicingQTL database (http://www.cancersplicingqtl-hust.com/#/) with the same filtering criteria [23]. Finally, we identified tag SNPs through the SNPinfo database (https://manticore.niehs.nih.gov/) and constructed the SNP-AS regulatory network [24].

### 2.6 CIBERSORT

CIBERSORT, a versatile computational method, could accurately estimate immune infiltration by RNA-seq, using LM22 data as a reference [25]. CIBERSORT was run according to code (https://rdrr. io/github/singha53/amritr/src/R/supportFunc_cibersort. R).

### 2.7 chemotherapeutic drugs prediction

The public pharmacogenomics database Genomics of Drug Sensitivity in Cancer (GDSC) was employed to predict the response to chemotherapeutic medicine [26]. The halfmaximal inhibitory concentration (IC50) was calculated by the ‘pRRophetic’ package in R.

### 2.8 Statistics

All statistical analyses were performed by R 3.5.3 and GraphPad prism 8.0.2. The threshold of significance was set at *P* < 0.05.

## 3 Results

### 3.1 Overview of AS events in the OV set

**Figure 1.**
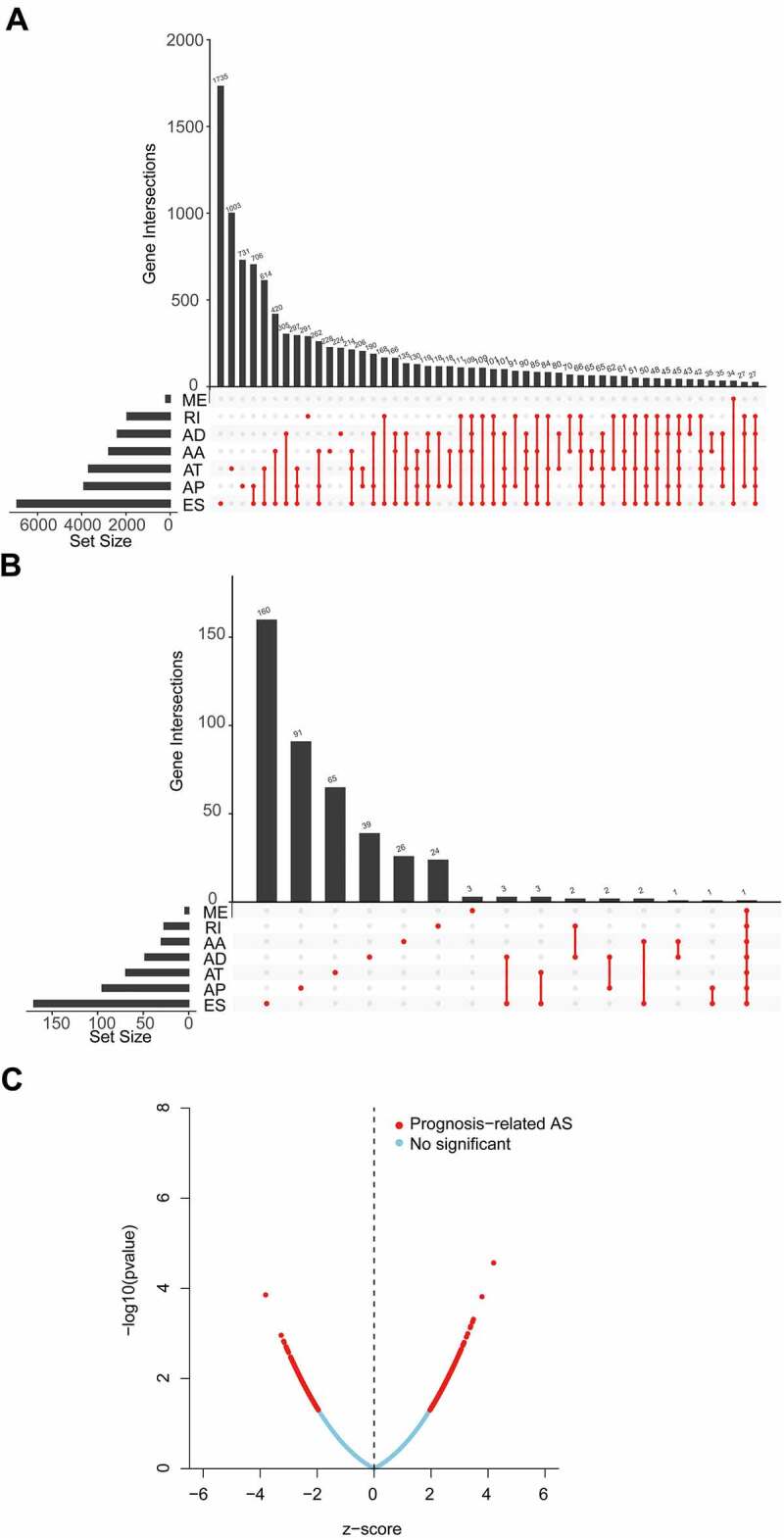
Overview of alternative splicing (AS) and prognosis-related AS events in ovarian cancer (OV).

AA, alternate acceptor; AD, alternate donor; AP, alternate promoter; AT, alternate terminator; ES, exon skip; ME, mutually exclusive exons; RI, retained intron. (a) The upset plot displayed seven types of AS events in OV. (b) The upset plot of prognosis-related AS events. (c) Volcano plot of prognosis-related AS events.

After integrating AS data, gene expression and clinical data, a total of 356 OV patients were included in the analysis. The detected AS events consisted of seven types: alternative acceptor site (AA), alternative donor site (AD), alternative promoter (AP), alternative terminator (AT), exon skip (ES), mutually exclusive exons (ME), and retained intron (RI). From the total AS event types, the most frequent events were ES events. In the current study, 48,049 AS events from 10,582 genes were identified, revealing that AS was a common process in the development of OV. Specifically, there were 19,251 ES events in 6931 genes, 9689 AP events in 3901 genes, 8453 AT events in 3691 genes, 4006 AA events in 2777 genes, 3497 AT events in 2389 genes, 2946 RI events in 1951 genes, 207 ME events in 201 genes (Figure 1A).

**Figure 2.**
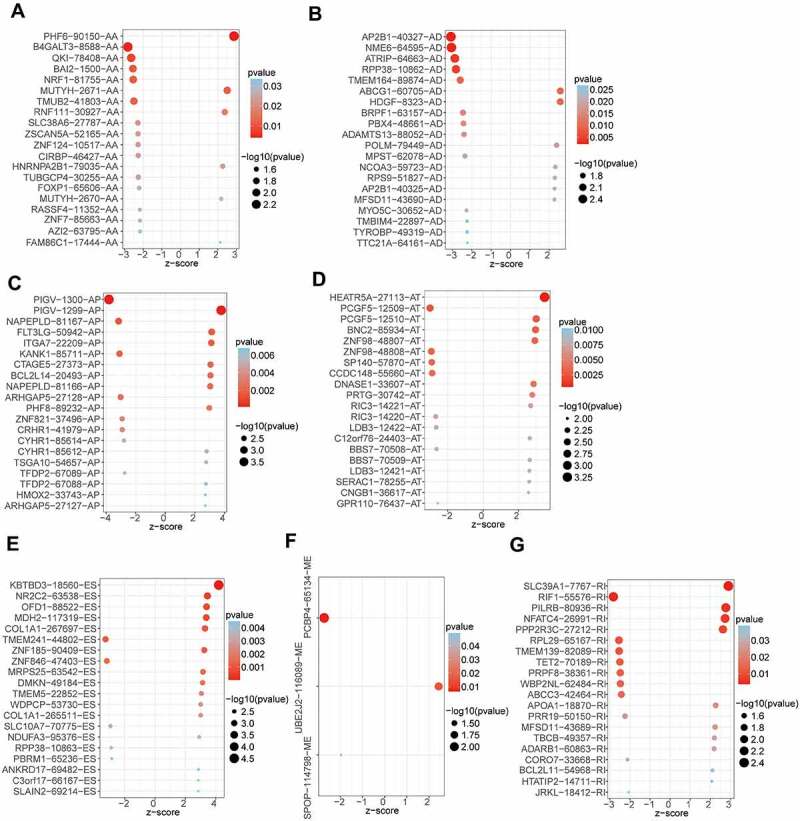
The top 20 most significant prognosis-related AS events in seven types of AS. (a) AA, (b) AD, (c) AP, (d)AT, (e)ES, (f)ME, (g) RI.

By univariate Cox analysis, 547 AS events of 422 genes were identified to be related to the OS of OV patients (Figure 1B, Supplementary Table S1). The 547 AS events comprised a total of 277 high-risk events and 270 low-risk events. The distribution of prognosis-related AS events was displayed in a volcano plot (Figure 1C). The top 20 ranked seven AS events associated with OS were shown in Figure 2.

### 3.2 AS prognostic risk model construction and validation

**Figure 3.**
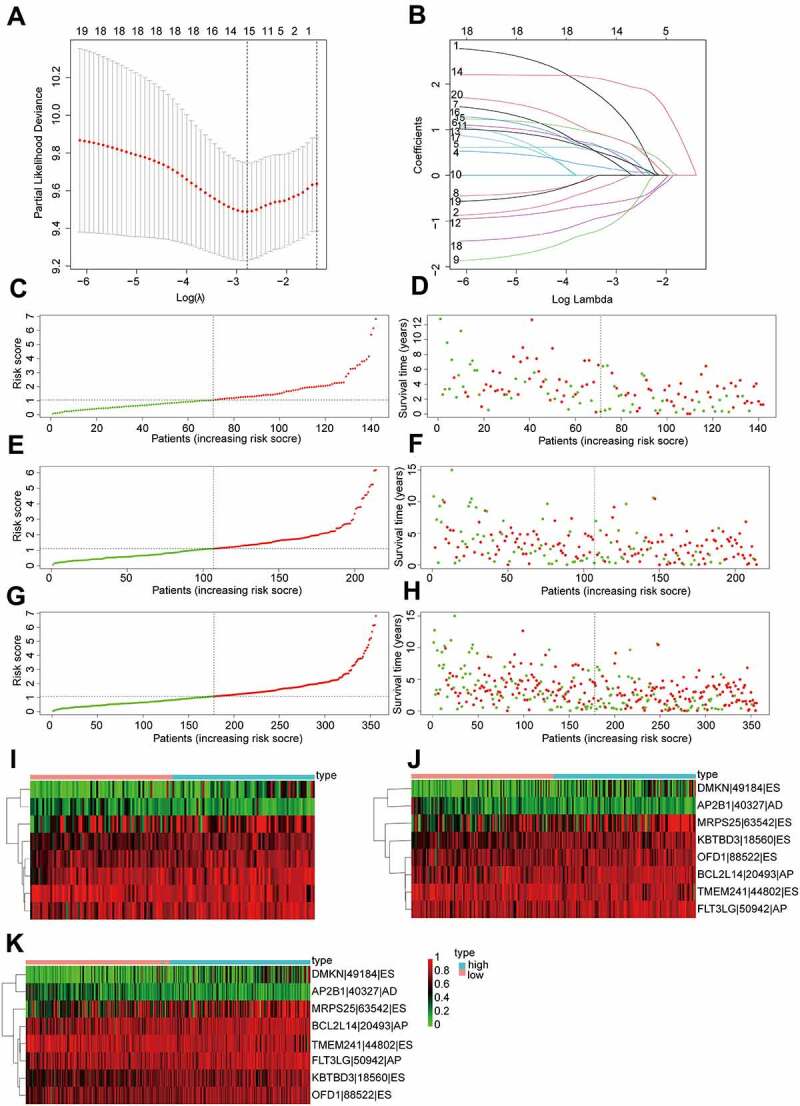
Construction and validation of AS prognostic risk model.

The top 20 prognosis-related AS events were selected as candidates. To avoid overfitting, the LASSO regression was employed to determine 12 AS events (Figure 3A-B). Subsequently, eight AS events (KBTBD3|18,560|ES, OFD1|88,522|ES, TMEM241|44,802|ES, MRPS25|63,542|ES, FLT3LG|50942|AP, DMKN|49184|ES, AP2B1|40,327|AD and BCL2L14|20,493|AP) were screened to construct a AS prognostic risk model via multivariate Cox regression analysis (Supplementary Table S2). The formula of risk score was calculated as follows: risk score = (2.80 * expression level of KBTBD3|18,560|ES) + (1.68 * expression level of OFD1|88,522|ES) + (−1.66 * expression level of TMEM241|44,802|ES) + (1.05 * expression level of MRPS25|63,542|ES) + (1.97 * expression level of FLT3LG|50942|AP) + (1.28 * expression level of DMKN|49184|ES) + (−1.91 * expression level of AP2B1|40,327|AD) + (1.90 * expression level of BCL2L14|20,493|AP). Then, patients were assigned into high-risk and low-risk groups based on the median risk score (training set and validation set). For candidate AS events, the detailed information of corresponding splicing pattern, living status as well as survival time ranked by the distribution of risk score was displayed in Figure 3C-K.

(a-b) LASSO Cox regression analysis of top 20 most significant prognostic AS events. (c-d) The distribution of risk score, overall survival (OS) and life status for the OV patients in the training set. (i) The expression heatmap of the most eight significant prognosis-related AS events in the training set. (e-f, j) Survival time, risk score distribution, and expression heatmap of AS events in validation set 1; (g-h, k) Validation set 2.

**Figure 4.**
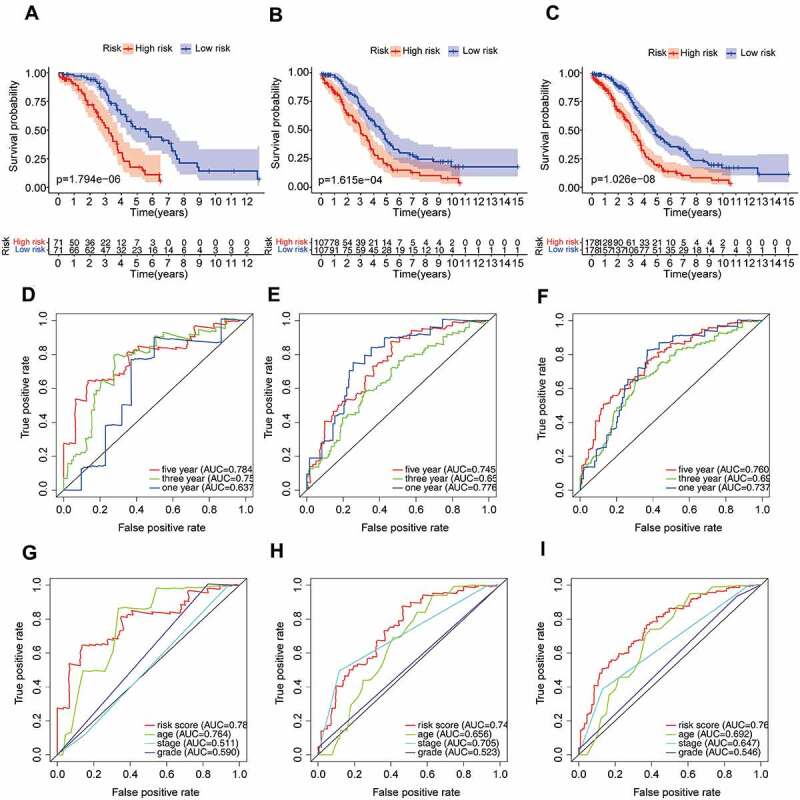
The risk score was closely related to the OV prognosis.

We further validated the relationship between risk score and prognosis by K-M survival analysis. The result suggested that the OV patients with low-risk score had a better prognosis than those with a high-risk score in the training set (*P* = 1.794e-06, Figure. 4A). Consistent with the training set results, patients with high-risk score tended to have poor prognosis (validation set 1, *P* = 1.615e-04, Figure 4B; validation set 2, *P* = 1.026e-08, Figure 4C). In addition, the predictive accuracy of the model was measured by the AUC. In the training set, the AUC at 1, 3, and 5 years were 0.637, 0.756, and 0.784, respectively (Figure 4D). In validation set 1, the AUCs of the prognostic risk model were 0.776, 0.655, and 0.745, respectively (Figure 4E). We found that the risk score also predicted the patient’s survival rate in the validation set 2, and the AUCs were 0.737, 0.693, and 0.760 (Figure 4F). Using the ROC curve, we further evaluated the predictive accuracy by computing the AUC of risk score, age, stage, and grade. Not only in the training set (Figure 4G), the AUC of risk score in the validation set (Figure 4H-I) was also much higher than that of other factors (age, stage, and grade). The above data demonstrated the powerful ability of the prognosis-related AS risk model for predicting prognosis.

Kaplan–Meier (K-M) curve (a), receiver-operating characteristic (ROC) analysis (d) and multiROC (g) of the risk score in the training set. (b, e, h) validaion set 1. (c, f, i) validaion set 2.

**Figure 5.**
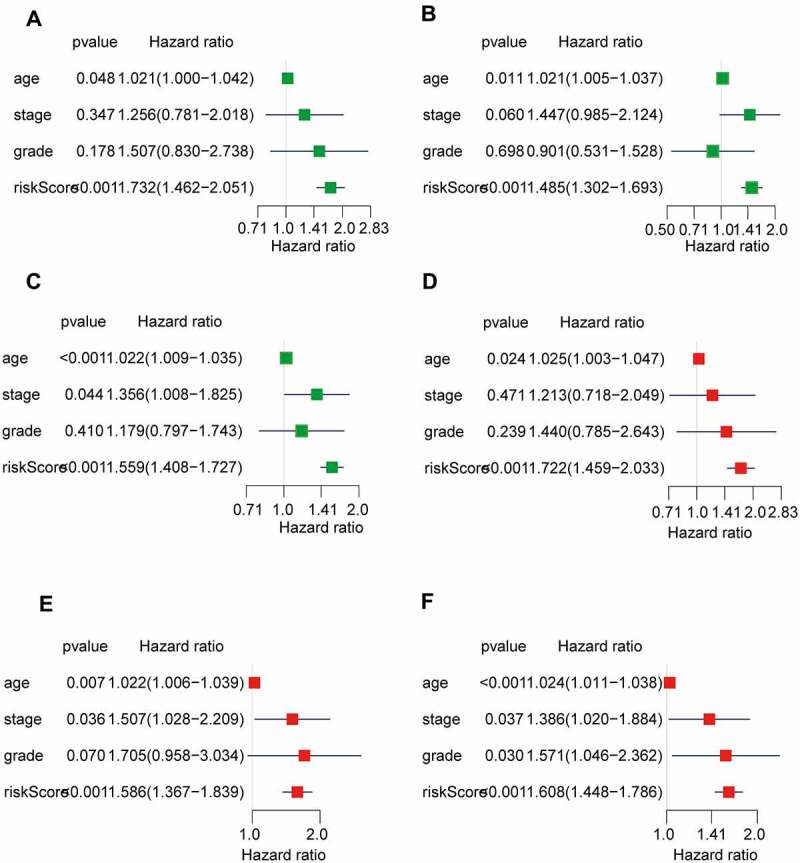
The risk score was an independent predictor of overall survival.

**Figure 6.**
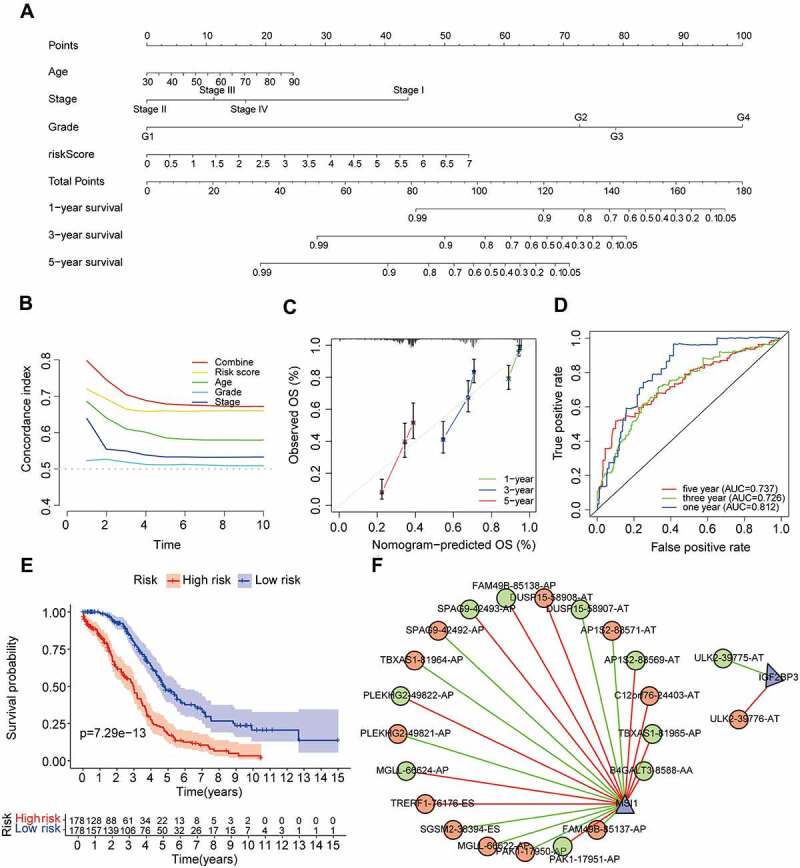
Establishment of OS nomogram.

The univariate and multivariate Cox regression analyses were performed to further assess whether the AS prognostic risk model was independent of other clinicopathological characteristics (age, stage, and grade). As expected, the risk score was a significant prognostic factor independent of other clinicopathological characteristics in the training (Figure 5A, Figure 5D) and validation set (Figure 5B-C, Figure 5E-F). As it is well known that the clinicopathological characteristics are important factors affecting the prognosis and treatment of patients with OV. Therefore, we reconstructed the clinically applicable model by integrating clinical variables and risk score through multivariate Cox regression [27]. Subsequently, we developed a nomogram model to predict the clinical outcome of OV patients and validated the predictive power using C-index, calibration plots, and ROC curves. These results demonstrated that the nomogram (C = 0.692) had a more accurate prediction than that of a single factor (Figure 6A, Figure 6B, Supplementary Table S3). Moreover, the calibration curves of 1-, 3-, and 5-year OS were very close to the ideal curve, showing good concordance between the nomogram predicted outcomes and the actual outcomes, suggesting appreciable reliability of the nomograms (Figure 6C). Similarly, the 1-, and 3-year AUCs of the clinically applicable model were 0.812, and 0.726, respectively, which were 7.5% and 3.3% higher than that of the risk score (Figure 6D). However, in the clinically applicable model, the 5-year AUC was slightly lower than the risk score (0.737 vs 0.760). Consistent with our previous results, OV patients with a high nomogram score had an extremely poor prognosis compared with patients with a low nomogram score (Figure 6E). Taken together, the clinically applicable model demonstrated a stable and robust ability to evaluate the prognosis of OV patients.

Univariate (a) and multivariate (d) Cox regression analysis of clinical features and risk score in the training set. (b, e) validaion set 1. (c, f) validaion set 2.

(a) Nomogram for predicting OS of OV. Concordance index (b) and calibration curve (c) of the nomogram. The ROC curve (d) analysis and K-M plot (e) of the clinically applicable model. (f) The interaction network between SF and AS events.

### 3.3 The SF-AS regulatory network and SNP-AS regulatory network

To explore the regulatory mechanism of AS events and SF, we analysed the correlation between SF expression and PSI values of 547 prognosis-related AS events. We identified a total of two key SF, of which, IGF2BP3 was associated with two AS events and MSI1 with 20 AS events (|R| > 0.3, *P* < 0.001, Figure 6F, Supplementary Table S4). It had been reported that SNPs were also participate in the AS of genes, so we constructed the SNPs and prognostic AS events regulatory network. Using the same screening criteria, we screened a total of 1247 SNPs associated with 32 AS events, and further obtained 156 tag SNPs through SNPinfo (https://manticore.niehs.nih. gov/) database (Supplementary Table S5).

### 3.4 Characteristics of high- and low-risk patients

**Figure 7.**
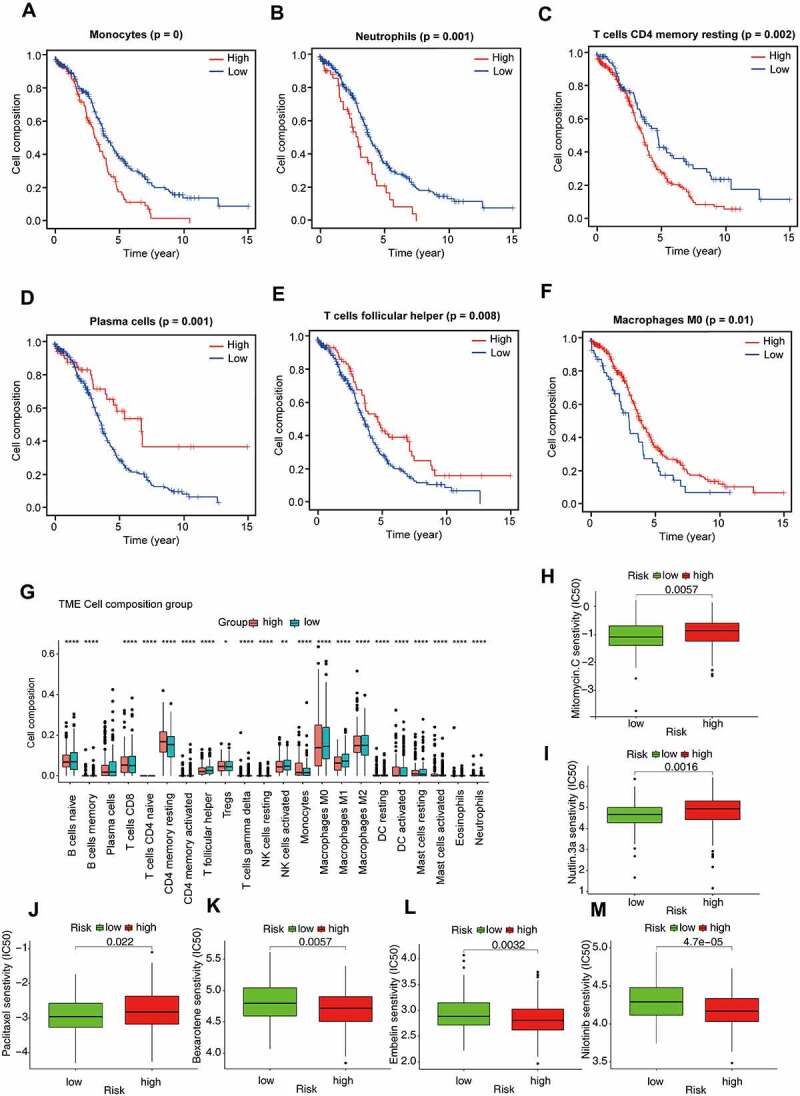
Immune cell infiltration and chemosensitivity of OV patients.

In order to estimate immune cell abundance between high- and low-risk groups, the CIBERSORT algorithm was used to estimate immune cell infiltration in TCGA-OV patients using gene expression data. We also analysed the relationship between the infiltrating of each cell type and OS via the K–M method. As shown in Figure 7D-F, patients with high plasma cells infiltration, T cells follicular helper infiltration, and Macrophages M0 infiltration had a better prognosis; However, high infiltration of monocytes, neutrophils, and T cells CD4 memory resting often predicted poor prognosis (Figure 7A-C, Supplementary Table S6). In addition, we noted significant differences in the proportion of immune cells between low- and high-risk OV samples. As revealed in Figure 7G, poor prognosis-related immune cells (including monocytes and T cells CD4 memory resting) in the high-risk group were more abundant than in the low-risk group. On the contrary, high proportions of immune cells associated with a good prognosis (including plasma cells and T cells follicular helper) were observed in the low-risk group. These results reflected multidimensional factors in tumour and immune microenvironment formation, which may provide new insights into poor prognosis in high-risk populations.

The K-M plot of immune cell in OV patients. Patients with high infiltration of monocytes (a), neutrophils (b), and T cells CD4 memory resting (c) often predicted poor prognosis. Patients with high plasma cells infiltration (d), T cells follicular helper (e) infiltration, and macrophages M0 (f) infiltration had a better prognosis. (g) Comparison of 22 immune cell infiltration between the high-risk group and low-risk group. (h-m) IC50 of chemotherapy drugs (including Mitomycin C, Nutlin 3a, Paclitaxel, Bexarotene, Embelin, and Nilotinib) in different groups.

### 3.5 Sensitivity of Chemotherapy Drugs Between groups

Radical surgery combined with adjuvant chemotherapy is the basic strategy for the treatment of ovarian cancer. Therefore, predicting the sensitivity of the two subgroups to chemotherapeutic agents might assist clinicians to formulate the optimal treatment plan. The response to chemotherapeutic drugs of OV patients was assessed based on the GDSC database. The results suggested that patients in the high-risk group were more sensitive to Bexarotene (*P* = 0.0057), Embelin (*P* = 0.0032) and Nilotinib (*P* < 0.001) than the low-risk group (Figure 7K-M). As revealed in Figure 7H-J, low-risk patients were more sensitive to clinical treatment (*P* = 0.0057 for Mitomycin C, *P* = 0.0016 for Nutlin 3a, *P* = 0.022 for Paclitaxel).

## 4 Discussion

OV is one of the most common malignancies of the female reproductive system. As the ovary is located deep in the pelvic cavity, the early lesions are not easy to be detected, which leads to that about 70% of OV patients being already in the advanced stage when diagnosed, making OV the leading cause of death from the gynaecological malignant tumour [28]. Currently, the treatment of OV is mostly limited to radical surgery and chemotherapy, which will prolong the recurrence interval but not be conducive to OS [29]. Therefore, establishing an effective prognostic prediction model is essential to guide the treatment of patients. AS, as one of the post-transcriptional regulatory mechanisms, removes introns from pre mRNA and joins exons to produce mature mRNA, which in turn increases protein complexity [20]. Previous studies have shown that AS plays an important role in regulating the growth, metastasis, drug resistance, recurrence of various tumours, including OV [15,30–33]. This study comprehensively explored the characteristics of the relationships among ovarian prognosis, AS, SF, SNPs, and tumour microenvironment, providing an important basis for further development of novel therapeutic strategies to improve the outcomes of patients with OV.

In this study, a total of 48,049 AS events involving 10,582 genes were identified, among which ES events were the most. Next, through univariate Cox regression analysis, prognosis-related AS events in OV samples were identified and 547 prognosis-related AS events were found in 422 genes. The top 20 genes most associated with survival were selected to construct the prognostic model, and eight AS events were identified by LASSO regression and multivariate COX regression analysis. The risk score of the samples was calculated according to the formula. Patients were divided into low-risk and high-risk groups based on the median risk score. The K-M plot showed that patients in the high-risk group tended to have a poor prognosis compared with those in the low-risk group (*P* = 1.794e-6). The ROC results also showed that the predictive ability of 1, 3, 5 years were 0.637, 0.756, 0.784, respectively, which belonged to the moderate strength of prediction. In addition, the predictive power of the risk score was also higher than that of age (AUC = 0.764), stage (AUC = 0.511), and grade (AUC = 0.590), indicating that risk score had important predictive significance for survival outcome of OV. Consistent with the training set, the same results were obtained in validation set 1 and validation set 2. In addition, we found that the model incorporating clinical variables improved the ability to predict prognosis compared with a single risk score.

AS events are closely related to TME, and immune cells, as an important component of TME, are key prognostic factors in patients with OV [34]. As in many other cancer types, the patients with good prognosis had plasma cell infiltration according to our findings [35]. Unlike other cancers, tumours rich in macrophage M0 suggested a better prognosis, which might be related to the direction of macrophage M0 differentiation and tumour heterogeneity [36]. In contrast, infiltration of monocytes and T cell CD4 memory suggested a poor prognosis. As expected, high-risk patients had higher abundances of monocytes and T cell CD4 memory, but lower abundances of plasma cells and macrophage M0. From this, we speculated that the different infiltration of immune cells in the AS prognostic risk model may result in the different prognosis of patients. Furthermore, according to the results of the GDSC database, patients in the low-risk group might benefit from chemotherapy drugs including Mitomycin C, Nutlin 3a and Paclitaxel. The difference is that patients in the high-risk group were more sensitive to Bexarotene, Embelin and Nilotinib.

To investigate the effect of upstream factors on AS, we analysed the correlation between 404 known SF and AS events. With *P* < 0.05, |R| > 0.3 as the threshold, this study found that MSI1 and IGF2BP3 were associated with multiple AS events. Previous studies had shown that RNA binding protein MSI1 was highly expressed in a variety of tumour tissues [37–39]. In OV, MSI1 acted as an oncogene, inducing phosphorylation of ERK protein, activating the expression of anti-apoptotic protein Bcl-2, and promoting migration and invasion of cancer cells [40]. Similar to previous studies, it was found in our study that MSI1 might regulate the variable splicing of multiple genes, or even have opposite regulation modes for the variable splicing of the same gene [9]. For example, MSI1 positively regulated PLEKHG2-49,822-AP, while negatively regulated PLEKHG2-49,821-AP. These results suggested that future studies should focus more on the expression of gene subtypes or different transcripts rather than the overall expression level of genes. As a member of the IGF2BPs family, a series of studies have shown that IGF2BP3 is associated with the proliferation and metastasis of colon cancer [41], bladder cancer [42], gastric cancer [43], and other malignant tumours [44,45]. In addition, IGF2BP3 can also participate in the regulation of mRNA stability and translation through the recognition of m^6^A sites, such as MYC, CDK6, or Cyclin D1 [41,46,47]. Liu and his team found that high expression of IGF2BP3 in clear cell ovarian carcinoma was associated with poor prognosis, and IGF2BP3 was also confirmed to promote the occurrence of clear cell ovarian carcinoma in vivo and in vitro [48]. Our study found that IGF2BP3 positively regulated ULK2-39,776-AT and negatively regulated ULK2-39,775-AT events. However, the specific mechanisms need to be further explored.

Regulation of AS can be controlled by genetic variants that can directly alter the splice site sequence. Guo et al found that SNP rs4383 was related to the splicing rate of exon 1.2 in MAFF, and compared with the C allele, SNP rs4384 G allele showed a higher splicing rate of exon 1.2 in MAFF [19]. A total of 1247 SNPs were identified to be involved in the regulation of 32 gene splicing. Snpinfo database (https://manticore.niehs.nih.gov/) was used to screen LD tag SNPs. Ultimately, a total of 156 SNPs were constructed to participate in the regulation of AS.

Although our study identified some important AS events, SF, and SNPs for the prognosis of OV, there were still some limitations in our study. The study was based on bioinformatics methods, which required experimental confirmation of research conclusions. In addition, model building and validation were based on TCGA data, and it would have been better to have another external validation dataset. Therefore, more work was warranted to further explore this molecular mechanism.

## 5 Conclusion

The study constructed and validated a prognosis-related AS risk model, and analysed the immune microenvironment and chemotherapeutic drug sensitivity in different groups. The upstream regulatory mechanisms of AS were explored from both splicing factors and genetic variation, which may provide new insights into the underlying mechanisms of OV development. However, the roles of AS events, SF, SNP, and the immune microenvironment in the tumorigenesis and progression of OV require further study to fully elucidate.

## Supplementary Material

Supplemental Material
